# The Efficacy of Functional Electrical Stimulation of the Abdominal Muscles in the Treatment of Chronic Constipation in Patients with Multiple Sclerosis: A Pilot Study

**DOI:** 10.1155/2016/4860315

**Published:** 2016-04-20

**Authors:** Christine Singleton, Abdel Magid Bakheit, Carla Peace

**Affiliations:** ^1^Birmingham Community Health Care Trust, West Midlands Rehabilitation Centre, Birmingham, UK; ^2^West Midlands Rehabilitation Centre, 91 Oak Tree Lane, Selly Oak, Birmingham B29 6JA, UK

## Abstract

Chronic constipation in patients with multiple sclerosis (MS) is common and the current methods of treatment are ineffective in some patients. Anecdotal observations suggest that functional electrical stimulation (FES) of the abdominal muscles may be effective in the management of constipation in these patients.* Patients and Methods*. In this exploratory investigation we studied the effects of FES on the whole gut transit time (WGTT) and the colonic transit time (CTT). In addition, we evaluated the treatment effect on the patients' constipation-related quality of life and on the use of laxatives and the use of manual bowel evacuation. FES was given for 30 minutes twice a day for a period of six weeks. Four female patients were studied.* Results*. The WGTT and CTT and constipation-related quality of life improved in all patients. The patients' use of laxatives was reduced. No adverse effects of FES treatment were reported.* Conclusion*. The findings of this pilot study suggest that FES applied to the abdominal muscles may be an effective treatment modality for severe chronic constipation in patients with MS.

## 1. Introduction

Chronic constipation resistant to medical treatment is common in patients with multiple sclerosis (MS). It has been reported in 43% of patients and the high prevalence does not correlate with disease severity or the presence of spinal pathology [1]. Chronic constipation in patients with MS is often associated with increased morbidity. For example, DasGupta and Fowler [2] have found that MS patients were 3 to 4 times more likely to be admitted to hospital with faecal impaction and megacolon than patients with other neurological conditions. Furthermore, chronic constipation also reduces the individual's quality of life and social participation. In a recent survey [3] 47% of MS patients reported that they were forced to make life style changes and 15% had to give up paid employment because of the impact of constipation on their normal activities of daily living. Severe chronic constipation may also affect patients' sex life and intimate relationships.

The traditional management of severe constipation with dietary modification, oral laxatives, suppositories, enemas, digital anal stimulation, and manual evacuation is usually only partially effective. Severe cases may require surgical treatment, such as subtotal colectomy and ileorectal anastomosis. In recent years sacral nerve stimulation has been shown to reduce constipation in patients with diminished rectal sensation, slow colonic transit time, and reduced urge to defecate [4–7] However, the implantation of the stimulator is an invasive surgical procedure that is associated with potentially serious complications [8]. Functional electrical stimulation of the abdominal muscles appears as a promising alternative.

Anecdotal reports by MS patients who were treated with FES for other indications suggest a significant increase in the frequency of bowel motions possibly as a result of this treatment. We have also studied a patient with severe chronic constipation in whom FES of the abdominal muscles increased the frequency of bowel evacuation and improved the patient's quality of life [9].

The aim of this study is to further evaluate the efficacy of FES of the abdominal muscles in the treatment of chronic functional constipation and to establish its effects on gut motility. Namely, we wish to establish whether FES of the abdominal muscles reduces whole gut and colonic transit time, reduces the need for laxatives, suppositories, enemas, digital anal stimulation, and manual bowel evacuation, and improves the patients' constipation-related quality of life.

## 2. Study Design 

This is a prospective exploratory pilot study. The study was approved by the relevant Ethics Committee and all patients gave an informed written consent to participate in the study.

### 2.1. Subjects

Adult patients with a diagnosis of multiple sclerosis who were able to eat and drink normally and who met Rome III criteria for functional constipation [10] and had constipation for at least three months were recruited. All patients had at least 2 of the following symptoms for 25% or more of defecations: straining, lumpy hard stools, sensation of incomplete evacuation, sensation of anorectal obstruction/blockage, manual manoeuvres to facilitate defecation, and less than 3 defecations per week for at least 3 months. In addition, they rarely had loose stools even when they used laxatives.

Patients with a history of irritable bowel syndrome, organic bowel obstruction or other bowel disease, or contraindications to FES (epilepsy, cardiac pacemaker in situ or other implanted electrical devices) were excluded.

The patient's usual diet, all medication, and other treatments were unchanged during the 6-week study period. Nonurgent MRI scans and X-ray investigations were not allowed until the SmartPill was excreted (usually by day 5).

### 2.2. Intervention

**Figure 1:**
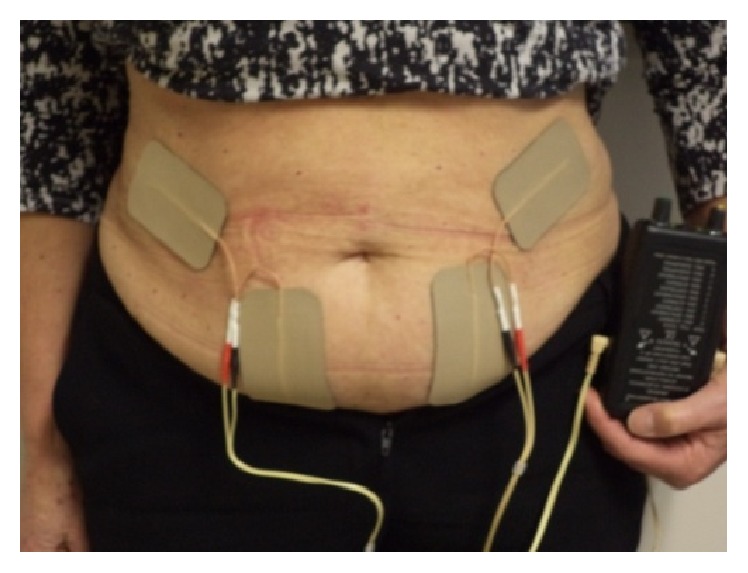


Functional electrical stimulation (FES) of the external oblique and transverse abdominis muscles at 40 Hz, 330 *µ* pulse width, and 40–50 mA was delivered with Microstim 2 (Odstock Medical Ltd., Salisbury, Wiltshire, UK, SP2 8BJ) using adhesive, rectangular (50 × 90 mm) silver carbon surface electrodes. Microstim 2 is a lightweight, portable battery-powered stimulator unit. The electrodes were placed over the muscle where the stimulation produced the strongest visible muscle contraction.  Figure 1 shows the site of electrodes placement.

Treatment was administered by the patients themselves or by their carers after training by the clinicians on electrodes placement and the use of FES. Treatment was initially given for 15 minutes twice daily for the first 2 days. Thereafter, the treatment session was increased to 30 minutes twice daily. The time of day for FES treatment was decided by the patients to coincide with their usual bowel routine. Treatment was given daily and continued for six weeks.

### 2.3. Study Outcome Measures

The primary outcome measure was the colonic and whole gut transit time. Secondary outcome measures were constipation-related quality of life and change in the need for laxatives, suppositories, enemas, digital anal stimulation, and manual bowel evacuation as recorded in a bowel diary.

Colonic and whole gut transit time were measured with the SmartPill wireless motility capsule (SmartPill Corporation, Buffalo, NY, USA) using a standard protocol [11]. The SmartPill is a small (13 mm × 26 mm) single use device containing sensors for the measurement of pressure, pH, and bowel transit time, as it passes through the gastrointestinal tract. The capsule is swallowed and the data are recorded on an external receiver. The test does not involve exposure to radiation or require admission to hospital.

The patients were asked to fast for 8 hours before the test. Drinking water, plain tea, or coffee without sweetener or cream was allowed. Patients were also allowed to take any prescribed medication except drugs that affect gastrointestinal motility. On the morning of the test the patients had a standardised meal known as the SmartBar and then swallowed the SmartPill. They fasted for the following 6 hours. The data collection started from the time of the ingestion of the SmartPill and continued until the SmartPill was expelled.

#### 2.3.1. Constipation-Related Quality of Life (C-R QoL)

C-R QoL was measured with the Patient Assessment of Constipation Quality of Life (PAC-QOL) questionnaire which has proven validity and reliability [12]. Briefly, the questionnaire consists of 28 items arranged under the following 4 subscales: physical discomfort, psychological discomfort, worries and concerns, and satisfaction. Each item is scored from zero (no problem) to 4 (very severe) and the total is divided by 28 (the number of the PAC-QOL items) to produce the mean overall score which can range from zero to four. The higher the PAC-QOL overall mean score, the poorer the quality of life.

#### 2.3.2. Bowel Diary

The patients were asked to record daily the frequency of defecation, consistency and size of stools (small or large), straining and the presence or absence of urge to defecate, and the sensation of incomplete bowel evacuation. The type and dose of laxatives, enemas, suppositories, and the frequency of digital anal stimulation and manual bowel evacuation were also recorded.

### 2.4. Assessments

Assessments were carried out on the first day of the study (baseline) and on completion of the trial at the end of week six. To monitor the safety of the procedure, the patients were asked to report adverse events by telephone and during scheduled clinic visits.

## 3. Results

Five female patients who met the inclusion criteria were recruited. One patient withdrew three weeks after the start of FES treatment due to a relapse of MS. The mean age of those who completed the study was 53.2 (range 45–58) years. The mean duration of MS since diagnosis was 22.7 (range 8–29) years.

As shown in Table 1, the whole gut transit time (WGTT) and the colonic transit time (CTT) were significantly reduced after FES in all patients. The posttreatment WGTT and CTT were close to the average values in healthy subjects of 73 and 59 hours, respectively.

The patients' constipation-related quality of life also improved. The results of PAC-QOL are shown in Table 2. In three patients the reduction in the mean overall PAC-QOL score after FES treatment was close to one point which is the critical threshold that is considered evidence of a meaningful clinical improvement [13], and in one patient it exceeded one point.

Review of the bowel diary confirmed improvement in the bowel habit of all patients at week six compared to baseline. Improvement in five out of the seven items was reported by two patients and in three items by the other patients (Table 3).

No adverse effects of treatment were reported by the patients or observed by the clinicians.

## 4. Discussion

The findings of the present pilot study have shown that FES applied to the abdominal muscles improved gut motility, as demonstrated objectively by reduction in the whole gut transit time, as well as the colonic transit time. Furthermore, all patients reported better constipation-related quality of life after the six-week treatment. The patients' use of laxatives had also reduced. There were no adverse effects of the FES treatment.

Although statistical analysis was not carried out because of the small sample size, there was clear improvement in the gut motility in all study patients. This is particularly important because of the objective nature of the evidence of reduced whole gut and bowel transit time and also because it was in agreement with the patients' reported improvement in quality of life. We used the SmartPill for the measurement of the whole gut and the bowel transit time. The safety, reliability, and validity of this method has been previously established [14].

The mechanism by which FES improved gut motility is not clear. A likely explanation is that it strengthened the abdominal muscles and increased the intra-abdominal pressure which enabled easier propulsion of the bowel contents. A direct effect on peristalsis is also possible.

FES is a safe and noninvasive method that can be administered by the patients themselves or by their carers in the home environment. The findings of the present pilot study suggest that FES may be an effective treatment modality for severe intractable functional constipation. A randomised controlled trial with the appropriate sample size is needed for the further evaluation of these findings.

## Figures and Tables

**Table 1:** Whole gut transit time (WGTT) and the colonic transit time (CTT) in hours before and after six weeks of treatment with functional electrical stimulation (FES) of the abdominal muscles. The percentage reduction of WGTT and CTT after treatment is shown in brackets.

Patient	Baseline WGTT	Post-FES WGTT	Baseline CTT	Post-FES CTT
1	124.02	81.22 (35%)	115.41	70.09 (39%)
2	118.29	78.53 (33%)	109.09	55.40 (49%)
3	141.44	70.04 (50%)	132.15	62.18 (53%)
4	201.27	146.23 (27%)	190.43	128.06 (32%)

**Table 2:** The mean score of the Patient Assessment of Constipation Quality of Life (PAC-QOL) questionnaire before and after FES treatment.

Patient	Baseline score	Posttreatment score	Score difference
1	1.14	0.71	0.43
2	1.86	1.00	0.86
3	1.86	0.71	1.15
4	1.57	0.86	0.71

**Table 3:** Change at week six from baseline in the patients' bowel habit.

Item of bowel diary	Patient 1	Patient 2	Patient 3	Patient 4
Straining to open bowels	+1	+1	+1	+1
Passing hard, lumpy stools	0	0	+1	+1
Incomplete bowel evacuation	+1	+1	+1	0
Feeling of “blockage” in bowels	0	+1	+1	+1
Use of manual evacuation of bowels	+1	0	0	0

Frequency of bowel motions	0	+1	+1	0
Number of loose stools per week	0	+1	0	0

Key: 0 = no change from baseline; +1 = significant improvement compared to baseline.

## Additional Points

The small sample size is the main limitation of this study. In addition, we relied on the patients' verbal reports of their diet during the study. The fibre content of a diet and the state of hydration have some effect on the frequency of bowel habit. Although our patients reported that their diet before the study and throughout the study period remained the same, a written record and analysis of the quantity and type of food and the amount of fluids taken by them would have provided more accurate information.
